# The Current Status and Future Prospects of Oncolytic Viruses in Clinical Trials against Melanoma, Glioma, Pancreatic, and Breast Cancers

**DOI:** 10.3390/cancers10100356

**Published:** 2018-09-26

**Authors:** Ibrahim Ragab Eissa, Itzel Bustos-Villalobos, Toru Ichinose, Shigeru Matsumura, Yoshinori Naoe, Noriyuki Miyajima, Daishi Morimoto, Nobuaki Mukoyama, Wu Zhiwen, Maki Tanaka, Hitoki Hasegawa, Seiji Sumigama, Branko Aleksic, Yasuhiro Kodera, Hideki Kasuya

**Affiliations:** 1 Cancer Immune Therapy Research Center, Graduate School of Medicine, Nagoya University, Nagoya 466-8550, Japan; brahimessa@med.nagoya-u.ac.jp (I.R.E.); bustositzel@med.nagoya-u.ac.jp (I.B.-V.); t-ichinose@med.nagoya-u.ac.jp (T.I.); smatsumu@med.nagoya-u.ac.jp (S.M.); ynaoe@med.nagoya-u.ac.jp (Y.N.); wu.zhiwen@med.nagoya-u.ac.jp (W.Z.); 2 Department of Surgery II, Graduate School of Medicine, Nagoya University, Nagoya 466-8550, Japan; d-morimoto@med.nagoya-u.ac.jp (D.M.); ykodera@med.nagoya-u.ac.jp (Y.K.); 3 Faculty of Science, Tanta University, Tanta 31527, Egypt; 4 Department of Transplantation and Endocrine Surgery, Graduate School of Medicine, Nagoya University, Nagoya 466-8550, Japan; miyajima@med.nagoya-u.ac.jp; 5 Department of Otolaryngology Graduate School of Medicine, Nagoya University, Nagoya 466-8550, Japan; tamgpad7042@gmail.com; 6 Takara Bio Inc., Kusatsu, Shiga 525-0028, Japan; tanakamy@takara-bio.co.jp; 7 Office of International Affairs, Graduate School of Medicine, Nagoya University, Nagoya 466-8550, Japan; hitoki@med.nagoya-u.ac.jp (H.H.); sumigama@med.nagoya-u.ac.jp (S.S.); branko@med.nagoya-u.ac.jp (B.A.)

**Keywords:** oncolytic viruses, clinical trials, melanoma, glioma, pancreatic, breast cancer clinical trials

## Abstract

Oncolytic viral therapy has been accepted as a standard immunotherapy since talimogene laherparepvec (T-VEC, Imlygic^®^) was approved by the Food and Drug Administration (FDA) and European Medicines Agency (EMA) for melanoma treatment in 2015. Various oncolytic viruses (OVs), such as HF10 (Canerpaturev—C-REV) and CVA21 (CAVATAK), are now actively being developed in phase II as monotherapies, or in combination with immune checkpoint inhibitors against melanoma. Moreover, in glioma, several OVs have clearly demonstrated both safety and a promising efficacy in the phase I clinical trials. Additionally, the safety of several OVs, such as pelareorep (Reolysin^®^), proved their safety and efficacy in combination with paclitaxel in breast cancer patients, but the outcomes of OVs as monotherapy against breast cancer have not provided a clear therapeutic strategy for OVs. The clinical trials of OVs against pancreatic cancer have not yet demonstrated efficacy as either monotherapy or as part of combination therapy. However, there are several oncolytic viruses that have successfully proved their efficacy in different preclinical models. In this review, we mainly focused on the oncolytic viruses that transitioned into clinical trials against melanoma, glioma, pancreatic, and breast cancers. Hence, we described the current status and future prospects of OVs clinical trials against melanoma, glioma, pancreatic, and breast cancers.

## 1. Historical Snapshots

It may come as a surprise that the treatment of cancer by using viruses was not the result of extraordinary experiments or clear-sighted theories. On the contrary, it stemmed from an observation by Dr. George Dock. In 1904, Dock reported the case of a patient with leukemia, whose leukocyte count decreased from 367,070 to 7500 over a period of two weeks, after an attack of what was presumed to be influenza [1]. Following this report, Levaditi noticed the growth and unusually long survival of the viruses in the vaccines administered to mice and rats with neoplasms [2]. Levaditi’s observations were confirmed by Rivers and Pearce, who concluded that the virus vaccine multiplied in a transplantable rabbit tumor of epithelial origin [3]. Throughout the 1920s and into the 1940s, viral biology was poorly understood, and there were few laboratory-based studies on viruses to investigate these previous observations. In addition, oncolytic viruses (OVs) were not studied in detail, because earlier works focused on virus growth in tumors, rather than studying the effects of these viruses on tumor growth [4]. In 1940, past observations inspired Pack to perform the first clinical trial in this field, in which an attenuated virus against melanoma led to a remarkable partial remission [5]. Subsequently, in 1949, another study showed an improvement in seven of 13 patients with Hodgkin’s Syndrome IX, who had progression of viral hepatitis for more than a month [6]. By the end of the 1940s and into the early 1950s, ex vivo virus cultivation was performed [7], which provided a better understanding of the proliferative capacity of cancer and normal cells [8].

In parallel with the advent of cell culture and viral manipulation, the concept of OVs was addressed by Moore in 1949, whose first known preclinical study in this field evaluated the ability of the Russian Far East Virus to inhibit the growth of five transplanted mouse tumors [9]. Other studies emphasized the anti-tumor effect of the Russian encephalitis virus in chicken tumors and sarcoma 180 [10,11], and cervical carcinoma [12] and the Semliki Forest virus in rabbit fibromas [13]. The most important discovery of this era was by Lindenmann and Klein, who demonstrated that post-oncolytic immunity was the result of enhanced humoral immunity against tumor cell antigens, via the secretion of immunoglobulin and cytotoxic antibodies [14]. In the 1970s and 1980s, several studies investigated the effects of a number of viruses on tumor regression in various leukemia models [15,16,17], Hodgkins’ disease [18,19], and Burkitt’s lymphoma associated with measles infection [20].

The revolution of recombinant DNA technology over the last 30 years made it possible to identify the essential genes for viral replication and the viral pathogenic mechanisms. The first studies to use genetically modified OVs were published in 1991 by Martuza et al. They developed herpes simplex virus (HSV) mutants (dlsptk) with depleted thymidine kinase or Infected cell protein (ICP) 34.5. These viruses were attenuated and demonstrated to be oncolytic, with selective replication in the diving cells of human glioma xenografts [21].

In the context of the recombinant DNA revolution, recent advances in viral cellular and genomic structure, as well as tumor development and immunology, have provided the main theme for using OVs as cancer therapy. In the last two decades, nine different families of viruses, including both DNA and RNA viruses, were successfully transitioned from preclinical studies into early randomized clinical-phase trials (Table 1). In this review, we will outline in detail the clinical trials of various OVs against melanoma, glioma, pancreatic, and breast cancers. In addition, we will discuss the progress of the clinical trial phases of each disease, and address the future of these trials. However, in this review, we selected the clinical trials that were completed and that reported the final results in the abstract or full paper for in four tumor types (melanoma, glioma, pancreatic, and breast cancer). There are other oncolytic viruses that proved their safety and efficacy in other tumor types.

## 2. Oncolytic Viruses and Melanoma Clinical Trials

Malignant melanoma is the most common cause of mortality from skin cancer worldwide [22]. Advanced (unresectable or metastatic) melanoma is associated with the lowest survival rate, because of its refractoriness to traditional therapies, such as chemotherapy and radiotherapy [23,24]. As there is a significant unmet therapeutic need in malignant melanoma, the interest in cancer immunotherapy has increased lately, and the Food and Drug Administration (FDA) approved four immunotherapeutic drugs for malignant melanoma treatment.

Three of these are immune checkpoint inhibitors, including two anti-PD1 antibodies named pembrolizumab (Keytruda^®^, Merck and Co., Inc., Kenilworth, NJ, USA) and nivolumab (Opdivo^®^, Bristol-Myers Squibb Company, New York, NY, USA), and an anti-CTLA4 antibody called ipilimumab (Yervoy^®^, Bristol-Myers Squibb Company) [25,26]. Moreover, in October 2015, talimogene laherparepvec (T-VEC, Imlygic^®^) became the first OV ever to be approved by the FDA and EMA [27]. However, a recombinant oncolytic adenovirus named H101 (Oncorine^®^, Shanghai Sunway Biotech) has been licensed by the China Food and Drug Administration (CFDA) for refractory head and neck carcinoma, in combination with chemotherapy, since November 2005.

### 2.1. Oncolytic Herpes Simplex Virus and Melanoma Clinical Trails

#### 2.1.1. Talimogene Laherparepvec (T-VEC, Imlygic^®^)

T-VEC is an attenuated herpes simplex virus-1 (HSV-1) that has been modified by the insertion of human granulocyte-macrophage colony-stimulating factor (GM-CSF) in place of both loci of the ICP34.5 gene, as well as by the deletion of the ICP47 gene. These modifications increased the selective replication within tumor cells, enhancing the tumor-specific immune response [28]. Phase I, II, and III clinical trials of T-VEC have been conducted against melanoma. The clinical phase I trial of T-VEC enrolled 30 patients with different cancer types, and among them were nine patients who were pathologically diagnosed with refractory or metastatic melanoma. Every two or three weeks, patients received intratumoral injections with doses of T-VEC, which ranged from 1 × 10^6^ to 1 × 10^8^ pfu/mL. Although there were no instances of complete response (CR) or partial response (PR) in this trial, as defined according to Response evaluation criteria in solid tumors (RECIST) 1.0, two patients achieved stable disease (SD), without any notable adverse events (AEs). Additionally, the lesions were flattened with evidence of necrosis [29]. This outcome supported the case for performing a clinical phase II trial of T-VEC in 50 patients with stage IIIc or IV melanoma. In this trial, patients received intratumoral T-VEC injections at a dose of 1 × 10^6^ pfu/mL (up to 4 mL) every two weeks, which was then increased to 1 × 10^8^ pfu/mL (up to 4 mL), for a total of 24 injections. The patients tolerated the therapy well, and developed only mild side effects such as a low-grade fever. In terms of outcomes, CR was achieved in eight patients, and PR was achieved in five patients, with a 26% overall objective response [30]. These promising results supported the use of T-VEC as an OV, and a phase III trial was therefore conducted in 436 patients with stage IIIb, IIIc, and IV unresectable melanoma. The patients were divided into two groups, 295 patients received intratumoral injections of T-VEC, and 141 patients received subcutaneous injections of recombinant GM-CSF. The objective response rate (ORR) was the primary endpoint and the overall survival rate was the secondary end point. The ORR and CR rates for T-VEC were 26% and 11%, respectively, compared to 6% and 1% for recombinant GM-CSF, respectively [31,32,33,34]. After proving its safety and efficacy, T-VEC was approved by FDA, in 2015, as the first OV for the treatment of melanoma patients [27]. Recently, the favorable safety profile and the ability to enhance the immune response made T-VEC an attractive candidate for use in combination with immune checkpoint inhibitors (anti-CTLA-4 (ipilimumab) and anti-PD-1 (pembrolixzumab). In a phase II study, T-VEC was combined with ipilimumab in 198 patients, with unresectable stages IIIB to IV melanoma. The patients were divided into two groups, a combination therapy group (T-VEC and ipilimumab (*n* = 98)) and a monotherapy group (ipilimumab alone (*n* = 100)). The patients received T-VEC in the first week with 4 mL (1 × 10^6^ pfu/mL), and in the third week the dose increased to 4 ml (1 × 10^8^ pfu/mL), repeating every two weeks. On the other hand, ipilimumab (3 mg/kg) was injected up to four doses every three weeks (in the monotherapy group, weeks 1, 3, 6, and 9, and in the combination therapy group, weeks 6, 9, 12, and 15). In the injected lesions, the combination therapy (T-VEC and ipilimumab) achieved an objective response in thirty-eight patients (39%) compared to eighteen patients (18%) who were treated with ipilimumab alone. On the other hand, the non-injected lesions were decreased in 52% of the patients in the combination treated group, compared to 23% of the patients in the monotherapy group. This trail demonstrated that T-VEC improved the efficacy of ipilimumab by increasing the objective response by nearly double when compared with the ipilimumab treatment alone [35]. Recently, another phase Ib investigated the combination therapy between T-VEC and immune checkpoint inhibitor, anti-PD-1 (200 mg pembrolizumab/every two weeks), in patients with advanced melanoma. In this trial, twenty-one patients received 4 mL (1 × 10^6^ pfu/mL) in the first week, and in the third week the dose was increased to 4 mL (1 × 10^8^ pfu/mL), repeating every two weeks. The objective response was 62% with 33% complete response rate, according to the immune related response criteria. An analysis of the tumor micro-environment revealed that infiltration of CD8 T-cells was increased with a high PD-L1 expression, as well as an IFN-g gene expression [36]. These results suggest that the combination of OVs and immune checkpoint inhibitors is better than either OVs alone or immune checkpoint inhibitors alone. To date, we can notice that two oncolytic viruses (HF10 and CVA-21) shift their strategy in phase II from a single therapy to a combination therapy with immune checkpoint inhibitors (discussed below in Section 2.1.2 and Section 2.1.3).

#### 2.1.2. HF10 (Canerpaturev—C-REV)

The approval of T-VEC marked the start of a new era of OVs for cancer treatment. Although T-VEC is the only OV that has been approved by the FDA and EMA so far, ongoing clinical phase II trials are investigating several OVs against melanoma, which will increase the chances that more OVs will be approved in the near future (Table 2). Among them, HF10 (canerpaturev) is an attenuated HSV-1 (the same family as T-VEC) with a natural deletion of UL56, and the latency-associated transcript (LAT) [37,38]. A phase I trial of HF10 enrolled 28 patients with refractory solid tumors consisting of cutaneous and/or superficial lesions. In the first stage, patients received single intratumoral injections of HF10 at doses of 1 × 10^5^, 3 × 10^5^, 1 × 10^6^, or 1 × 10^7^ median tissue culture infective dose (TCID_50_). In stage 2, the dosage of HF10 was increased from four injections of 1 × 10^6^ to 1 × 10^7^ TCID_50_. Treatment-related adverse events (AEs) were observed in 34.6% of patients, all of which were mild. Regarding the tumor response, SD was achieved in 66.7% (6/9) of the melanoma patients, and 13.3% (2/15) of the head and neck cancer patients. Neither CR nor PR were observed at the end of study, three months after the last injection of HF10. Interestingly, however, three melanoma patients showed a delayed and durable response after the end of the study [39]. The safety and tolerability of HF10 monotherapy was demonstrated in this phase I trial. The tumor response results supported the further investigation of HF10 in a phase II trial in melanoma patients. This trial of HF10 combined with ipilimumab (anti–CTLA-4) enrolled 46 patients (efficacy was evaluable in 44), and showed a best overall response rate as evaluated by immune-related (ir) response criteria: (irCR 18%; irPR 23%) of 41%, with a clinical benefit rate of 68% (irCR 18%, irPR 23% and irSD 27%). Combination therapy prolonged the median overall survival time by about 26.3 months (not reached), and enhanced the efficacy of ipilimumab in melanoma patients [40,41]. Of important note, regarding HF10, whose clinical development is in progress in Japan, the new Pharmaceuticals, Medical Devices, and Other Therapeutic Products Act (PMD Act, 2014) allows for the conditional and time-limited approval of regenerative medical products, including OVs, based on demonstrated safety and estimated efficacy in small-scale clinical trials. Under this new legislation, a new drug application for HF10 will likely be submitted soon after the phase II trial.

#### 2.1.3. Coxsackievirus A21 (CAVATAK^®^) and Melanoma Clinical Trails

Another natural OV named CVA21 (coxsackievirus A21, CAVATAK^®^), which belongs to the *Picornaviridae* family, was also studied in melanoma patients. CVA21 mainly binds to the N-terminal domain of intercellular adhesion molecule-1 (ICAM-1). As a consequence, CVA21’s effect depends on the expression of ICAM-1 on cancer cells [42]. In theory, as ICAM-1 is overexpressed on melanoma cells relative to normal cells, CVA21 should selectively replicate within tumor cells [43]. CVA21 demonstrated its safety in a phase I trial against melanoma [44,45]. A phase II trial of CVA21 enrolled 57 patients with unresectable stage IIIc-IVM1c melanoma. The patients received intratumoral injections of CVA21 (3 × 10^8^ TCID_50_) on days one, three, five, and eight, and then every three weeks for a total of six injections. The durable response rate was 28.1% (16 of 57 patients), and 38.6% (21 of 57 patients) of the patients had immune-related progression-free survival (irPFS) six months after the treatment [46]. Parallel to these findings, the preliminary results of the phase Ib study (ASCO2017) evaluated the combination therapy of CVA21 with ipilimumab in patients with advanced melanoma. Twenty-six patients (13 of 26 previously treated with anti-PD-1 antibody) received 3 × 10^8^ TCID_50_ CVA21 on days 1, 3, 5, 8, and 22, followed by six injections of ipilimumab (3 mg/kg), which began on day 22. The overall response rate was 38% and 88% disease control rate (DCR): (complete response (CR), partial response (PR), and stable disease (SD)) [47,48]. In the near future, the final results of CVA21 against melanoma will give us a clearer insight into the efficacy of CVA21 as a monotherapy, as well as in combination therapy with ipilimumab, for melanoma patients.

### 2.2. Pelareorep (Reolysin^®^) and Melanoma Clinical Trails

Pelareorep (Reolysin^®^) is a non-enveloped, double-stranded RNA virus that depends on the Ras mutation to produce selective replication in tumor cells [49,50]. In a phase I trial, the adequate intravenous delivery of pelareorep was demonstrated with the administration of 3 × 10^10^ TCID_50_ for five consecutive days, every four weeks; this dose was defined as the maximum tolerated dose. The study showed that the administration of the virus via intravenous induced highly neutralizing antibodies (neutralizing anti-reovirus antibody (NARA) response). The NARA response might be an obstacle for efficient viral replication. Six melanoma patients showed no objective response. Moreover, viral shedding was not observed, except at 5 or 15 days in a small minority of patients [51]. One drawback of this trial is the fact that, while pelareorep may be an appropriate therapeutic approach in tumors with oncogenic Ras mutations, the presence of these mutations was not investigated in the study population. A phase II study was also conducted with pelareorep in 21 patients with metastatic melanoma. The patients received intravenous injections of 3 × 10^10^ TCID_50_ on days one to five in each 28-day cycle (the same schedule as in the phase I trial). The overall survival rate was 168 days, with no objective response. As a consequence, the use of pelareorep as a monotherapy in melanoma treatment did not proceed to the second stage, and was considered to be more effective in combinatorial strategies [52]. In this regard, a recent phase II study administered pelareorep, carboplatin, and paclitaxel to patients with advanced melanoma. On the first day, 14 patients were treated with paclitaxel (200 mg/m^2^; 3 h intravenous infusion), followed by carboplatin (6 mg/mL; 30 min intravenous infusion) and posteriorly with pelareorep (3 × 10^10^ TCID_50_; 1 h intravenous infusion). Later, only the pelareorep dose was repeated for five consecutive days. The treatment cycle was repeated every 21 days for up to eight cycles. A previous trial of a paclitaxel/carboplatin combination against melanoma showed overall response rates of 19–26%. The triple combination of pelareorep/paclitaxel/carboplatin showed a 21% objective response rate (ORR), without any complete responses observed. At this point, the combination trial met the predetermined efficacy target for its first stage [53]. This trial revealed important insight regarding the strategies of combination therapies with OVs.

### 2.3. Vaccinia-GM-CSF (Pexa-Vec, Formerly Named JX-594) and Melanoma Clinical Trails

Vaccinia-GM-CSF, named pexastimogene devacirepvec (Pexa-Vec, formerly named JX-594), was used to treat melanoma patients in a clinical trial in 1999. Seven immunocompetent patients received intratumoral injections of escalating doses, ranging from 1 × 10^4^ to 8 × 10^7^ pfu, twice a week for six weeks. The trial demonstrated PR in one patient, complete remission in one patient, and a mixed response in three patients [54]. Afterwards, vaccinia-GM-CSF was named JX-594. In a phase I study, ten patients with stage IV melanoma were intratumorally injected with JX-594 (1 × 10^8^ pfu) every week, for a total of nine injections. Two patients withdrew from the trial after the second dose [55]. Six patients showed a significant induction of GM-CSF, resulting in a 25–300% increase in neutrophil levels. In addition, JX-594 replicated well in tumor cells, with lymphocyte infiltration leading to tumor necrosis.

### 2.4. Ongoing Melanoma Clinical Trails

Currently, phase I trials for another three OVs are ongoing against melanoma, namely, ONCOS (adeno ∆24-RGD-GM-CSF insertion) (NCT03003676), herpes virus OrienX010 (NCT03048253), and ICOVIR-5 (NCT01864759) (Table 2). 

## 3. Oncolytic Viruses and Clinical Trials in Malignant Glioma

Malignant glioma is the most highly invasive primary brain tumor [56]. The conventional treatment options for brain tumors are surgical resection, radiotherapy, and/or chemotherapy, depending on the tumor size, location, and pathological diagnosis. The treatment outcome is generally poor [57]. Several oncolytic viruses proved their safety and efficacy in several preclinical studies. Here, we report the oncolytic viruses that successfully transferred into the clinical trials.

### 3.1. Oncolytic Herpes Simplex Virus and Glioma Clinical Trials

Three oncolytic HSV-1 strains (HSV1716, G207, and G47∆) have completed the phase I trial in glioma patients. Currently, dose-escalation phase I studies of HSV-1-M032 and HSV-1-QNestin34.5 are being done to evaluate the safety and efficacy of these OVs in malignant glioma patients [58]. The following oncolytic herpes simplex viruses were attenuated by the deletion of ICP34.5, which confers neurovirullence.

#### 3.1.1. HSV1716 (Seprehvir^®^) and Glioma Clinical Trials

HSV1716 (Seprehvir^®^) was derived from HSV-1 strain 17 and attenuated by a mutation of ICP34.5 (deletion of 759 bp) [59]. A clinical phase I trial of HSV1716 enrolled nine patients with relapsed malignant glioma. The patients were intratumorally inoculated with up to 1 × 10^5^ pfu of HSV1716. The treatment was tolerated well, with no induction of HSV encephalitis. Four patients survived for 24 months after the HSV1716 treatment [60]. Another clinical study of HSV1716 enrolled 12 patients with malignant glioma. In this study, patients received a single intratumoral dose of HSV1716 (1 × 10^5^ pfu). After four to nine days of treatment, in 10 of the 12 patients, viral DNA was detected within the tumors. Five patients showed an immunological response through detectable changes in HSV-specific IgG and IgM. Three patients were clinically stable for an average of two years after treatment with surgery plus HSV1716 [61,62]. Recently, a phase II trial involving HSV1716 against recurrent glioma in children was completed, but the results are still pending (NCT02031965).

#### 3.1.2. G207, G47, and Glioma Clinical Trials

G207 is derived from wild-type HSV-1 strain F. G207 was attenuated by a deletion in both copies of *ICP*34.5 and an inactivating of ICP6 by insertion of the *Escherichia coli lacZ* gene in *UL39* [63]. A phase I trial of G207 enrolled 21 patients with glioblastoma. The following G207 doses were administered to three patients, 1 × 10^6^, 1 × 10^7^, 3 × 10^7^, 1 × 10^8^, 3 × 10^8^, 1 × 10^9^, and 3 × 10^9^ pfu each. The trial demonstrated that G207 inoculation into brain tumors was safe and did not lead to HSV encephalitis. Some patients had complications that are frequently associated with brain tumors, such as mental status changes, as well as dysphasia due to tumor edema, surgical trauma, or viral toxicity. This trial had some limitations, for instance, the lack of any evidence of viral replication [64]. A phase Ib of G207 enrolled six patients with glioma. The patients received two doses of G207 for a total 1.15 × 10^9^ pfu. All of the patients showed stable disease after one month of G207 inoculation, without any significant AEs or the development of encephalitis. The median survival was 6.6 months after G207 administration. Viral RNA encoding HSV DNA polymerase (pol) was detected in the tumor samples of all of the patients, without viral shedding to the blood or saliva [65,66]. In a recent phase I trial, nine patients received one dose of G207 (1 × 10^9^ pfu) 24 h prior to a single 5 Gy radiation dose. All of the patients tolerated G207 well. The median survival in this trial was 7.5 months after treatment with G207 [67]. Later, G47∆ was created by adding another deletion of ICP47 to G207 [68]. A phase I of G47∆ against glioma was completed, but the results have not been reported. Currently, a phase II study of G47∆ is ongoing in patients with residual or recurrent glioblastoma in Japan [69].

### 3.2. Oncolytic Adenoviruses and Glioma Clinical Trials

ONY015 oncolytic adenovirus was evaluated in a clinical phase I trial that enrolled 24 patients with malignant glioma. Four groups of patients received different doses of ONYX015 (1 × 10^7^ pfu in cohort one; 1 × 10^8^ pfu in cohort two; 1 × 10^9^ pfu in cohort three; and 1 × 10^10^ pfu in cohort four) in a total of 10 tumor sites. The patients showed no severe AEs, but neither antitumor effect was demonstrated, as the median survival was of only 6.2 months [70]. Another promising oncolytic adenovirus called DNX-2401 (Delta-24-RGD adenovirus) increases the viral replication selectivity through the deletion of 24 bp in EIA and the insertion of the RGD-motif into the fiber H-loop, which enables the virus to use αvβ3 or αvβ5 integrins (enriched on tumor cells) to enter the tumor cells. Recently, a dose-escalation trial for DNX-2401 (Delta-24-RGD adenovirus) in patients with recurrent malignant glioma was completed. In this trial, the patients were enrolled in two groups, group A, which included twenty-five patients receiving a single dose of DNX-2401 (1 × 10^7^–3 × 10^10^ vp), followed by monitoring for the toxicity and treatment response; and group B, which included twelve patients who were injected with DNX-2401 (1 × 10^7^–3 × 10^10^ vp) into multiple sites through implanted catheter, then the injection of a second dose of DNX-2401 (1 × 10^7^–3 × 10^10^ vp) on day 14 after the tumor resection, along with a catheter, to acquire post-treatment specimens. Remarkably, in group A (single treatment), five patients (20%) survived more than three years. Moreover, three patients out of the five patients had a 95% reduction in the tumor sizes. In group B, the post-treatment tumor specimens revealed that DNX-2401 was well replicated and induced tumor cell lysis, as well as the infiltration of CD8+ T Cells and the downregulation of transmembrane immunoglobulin mucin-3 [71,72]. This data indicates that Delta-24-RGD can achieve a durable complete response in some subsets of glioma patients. Finally, these results suggested the oncolytic effects of DNX2401, through the direct cell lysis and through enhancing the immune system, may be promising in the future for DNX2401 for combining with other immune modulatory therapies. Until now, DNX-2401 has two ongoing phase I trials; phase I study investigates the combination therapy between DNX2401 and temozolomide (NCT01956734). A multicenter phase Ib trial investigates DNX-2401 as a monotherapy or DNX-2401 in combination with interferon-γ in recurrent glioblastoma (NCT02197169).

### 3.3. Pelareorep (Reolysin^®^) and Glioma Clinical Trials

A phase I trial of pelareorep enrolled 12 patients with grade III and IV recurrent malignant glioma, with escalating doses from 1 × 10^7^ to 1 × 10^9^ TCID_50_. Pelareorep was injected into three intratumoral sites. Grade I and II toxicities were observed, without reaching the maximum tolerated dose. Tumor progression was observed in 10 patients, and stable disease was observed in one patient, with a median survival of 21 weeks, while only one patient had a survival of 54 months [73]. In another multicenter study, pelareorep was intratumorally injected in 15 patients with recurrent malignant glioma, at five different dose levels (1 × 10^8^ to 1 × 10^10^ TCID_50_). Interestingly, pelareorep was delivered through a method known as “convection enhanced delivery”, whereby pelareorep infusion into the tumor location was performed via catheter, with a continuous low pressure. Grade I and II side effects, such as seizure, were noticed in some patients. One patient experienced convulsions, a grade III AE. However, evidence of anti-tumor efficacy was observed in a few patients. Stable disease was observed in two patients, PR in one patient, and progressive disease (PD) in 12 patients. The median survival in this trial was 20 weeks, compared with 21 weeks in a previous trial [74]. Therefore, the pelareorep outcomes against glioma may be improved when combined with other therapeutic drugs such as bevacizumab (Table 3).

### 3.4. Newcastle Disease Virus and Glioma Clinical Trials

A phase I/II trial of Newcastle disease virus (NDV), HUJ strain, enrolled 11 patients with recurrent glioblastoma. The patients were divided into two groups, as follows: The first group received intravenous NDV-HUJ doses that ranged from 1 × 10^8^ to 11 × 10^9^ EID_50%_ weekly, with a total of three cycles of treatment. The second group received intravenous 11 × 10^9^ EID_50%_ weekly, for a total of three cycles. The intravenous treatment was well tolerated. Grade I and II side effects such as fever, seizures, stupor, syncope, headache, abdominal pain, and hypertension were observed in five patients. The median survival was 33.2 weeks. Of note, CR was observed in one patient [75]. However, since 2005, no other studies have evaluated the NDV-HUJ virus in glioblastoma patients.

### 3.5. ParvOryx and Glioma Clinical Trials

Recently, a trial of Parvovirus H-1 (ParvOryx) enrolled 18 patients with malignant glioma. ParvOryx was administered on two different schedules, as follows: The first used a single dose of ParvOryx injected intratumorally, with re-injection on day 10 after surgical resection. The second included five doses of ParvOryx injected intravenously, with intratumoral re-injection on day 10, after surgical resection. ParvOryx was well tolerated without a dose-dependent toxicity, regardless of the administration schedule. This trial was very important as it was the first to prove that ParvOryx could cross the blood–brain/tumor barrier in both directions. Moreover, ParvOryx induced the infiltration of T-cells, microglia, and macrophages within tumors, with a low number of infiltrated regulatory T-cells (Treg). This is the first study to provide a detailed description of the tumor immune response in glioma patients after treatment with an OV, through the detection of Tregs and several activation markers for immune cells, including perforin, granzyme B, interferon-γ, interleukin 2 (IL-2), CD40L, and CD25 [76].

### 3.6. Poliovirus and Glioma Clinical Trials

PVSRIPO is an attenuated poliovirus type 1 (Sabin), and its cognate internal ribosome entry site is replaced with the internal ribosome entry site of human rhinovirus type 2. PVSRIPO mainly binds to the poliovirus receptor CD155. CD155 is widely expressed in solid tumors. A dose-escalation trial was designed for five doses levels (1 × 10^8^ TCID_50_/one patient, 3.3 × 10^8^ TCID_50_/one patient, 1 × 10^9^ TCID_50_/one patient, 3.3 × 10^9^ TCID_50_/two patients, and 1 × 10^10^ TCID_50_/four patients). This escalation study revealed that 1 × 10^10^ TCID_50_ is the dose limiting due to one patient had a grade 4 intracranial hemorrhage. In the dose expansion phase, six patients received 3.3 × 10^8^ TCID_50_, 31 patients received 5 × 10^7^ TCID_50_, and 15 patients received 1 × 10^7^ TCID_50._ However, viral neuropathogenicity was not observed in the treated patients. A grade 3 or higher adverse event was observed in 19% of the patients, which related to PVSRIPO. Hence, a 5 × 10^7^ TCID_50_ dose level was determined for future phase II. Interestingly, the overall survival was 21%, which is higher than the rate among historical controls [77]. In conclusion, several OVs have clearly demonstrated both safety and promising efficacy in phase I trials in glioma patients. Consequently, several of these OVs will most likely be investigated in phase II trials in the near future.

### 3.7. Ongoing Glioma Clinical Trials

Clinical trials of three OVs have been initiated and are enrolling patients with glioma, measles virus CEA (NCT00390299), Toca 511 (NCT02576665), and vaccinia virus TG6002 (NCT03294486) (Table 3).

## 4. Oncolytic Viruses and Pancreatic Cancer

Pancreatic cancer is one of the major causes of cancer-related deaths worldwide [78], with the majority of these caused by pancreatic adenocarcinoma, the most prevalent subtype [79]. Pancreatic ductal adenocarcinoma (PDAC) does not express neoantigens, which consequently limit the priming of T-cells and the overall immune response [80]. Moreover, the pancreatic tumor microenvironment is enriched with immunosuppressive factors such as Tregs, M2 tumor-associated macrophages, IL-10, and transforming growth factor- beta (TGF-β). In addition, pancreatic tumors are primarily composed of fibrotic tissue and stellate cells, which together act as a natural physical barrier that renders the extracellular matrix impenetrable [81]. The combination of these factors limits the efficacy of both chemotherapy and immunotherapy [82,83,84]. Several chemotherapeutic drugs had been approved against PDAC, such as gemcitabine, folfirinox, and nab-paclitaxel, but all have been associated with severe side effects and only modest survival rates [85,86]. However, immune checkpoint inhibitors have shown significant efficacy against other types of cancer, such as melanoma. The efficacy of these drugs is still under investigation, and clinical trials against pancreatic cancer are ongoing [87]. On the other hand, several OVs were investigated in preclinical pancreatic models, either alone or in combination with chemotherapeutic drugs such as gemcitabine. Furthermore, some OVs are already under investigation in phase I and II clinical trials. Here, we will review the completed clinical trials of different OVs against pancreatic cancer (Table 4).

### 4.1. Oncolytic Adenoviruses and Pancreatic Clinical Trials

Oncolytic adenoviruses have been developed and investigated in preclinical pancreatic models. So far, the results of the phase I and II trials in PDAC patients have been reported only for the oncolytic ONYX-015 adenovirus. ONYX-015 (dl1520) is an E1B-55 kDa gene-deleted virus that replicates selectively in p53-mutated tumor cells [88]. A phase I trial of ONYX-015 enrolled 23 patients with unresectable pancreatic cancer. The patients were administered ONYX-015 (1 × 10^8^ to 1 × 10^11^ pfu) every four weeks via computed tomography (CT)-guided injection. All of the patients tolerated ONYX-015 without significant complications, except for one, who experienced transient pancreatitis [89]. High levels of neutralizing antibodies were observed in all of the patients who received treatment; moreover, viral replication was not detected by PCR in the blood or by fine needle aspirates. The authors highlighted the following two possible limitations regarding the detection of viral replication within tumors: (1) the aspirated samples from tumors were mainly composed of necrotic cells with few viable cells, and (2) the physical barriers of tumors limited viral replication. Overall, an objective response was not observed in this trial [89]. However, the results of this phase I trial supported the safety of ONYX015 and suggest that it is reasonable to test it as part of combination therapy. A phase II trial of ONYX015 combined with gemcitabine enrolled 21 patients with advanced and metastatic pancreatic adenocarcinoma [90]. The majority of patients received 2 × 10^11^ pfu ONYX015 via endoscopic ultrasound (EUS) guidance over eight weeks, with three patients receiving only 2 × 10^10^ pfu, while gemcitabine (intravenous, 1000 mg/m^2^) was administered during the final four weeks. A clinical response was not noted after treatment with ONYX-015 alone. However, after the administration of gemcitabine, six patients demonstrated SD, two showed partial regression, and two had a minor response; in all of these cases there were no severe side effects. Although this trial proved the significance of ONYX015 administration with EUS guidance, it did not overcome the problems of phase I, such as viral replication and the elevation of neutralizing antibodies, which represent a major challenge regarding treatment using OVs. In addition, ONYX015 showed limited efficacy, even in combination with gemcitabine [90]. Two other oncolytic adenoviruses, VCN-01 and LOAd703, are currently being evaluated in ongoing phase I trials, either alone or combined with nab-paclitaxel/gemcitabine in patients with metastatic pancreatic adenocarcinoma (NCT02705196, NCT02045589, and NCT02045602).

### 4.2. Oncolytic Herpes Simplex Viruses and Pancreatic Clinical Trials

Seven oncolytic herpes simplex viruses are currently under investigation in multiple phase II clinical trials in different types of solid tumors (Table 1). Only, HF10 and T-VEC were evaluated in clinical trials in pancreatic cancer patients (Table 4). In the investigator-initiated phase I trial of HF10 monotherapy, eight patients with pancreatic ductal carcinoma were enrolled and treated. HF10 (1 × 10^5^ to 1 × 10^6^ pfu) was intratumorally injected for three consecutive days, with the first dose injected during laparotomy. An intratumoral catheter was also inserted during surgery, to inject the other two doses of HF10. All of the patients tolerated the treatment well. without any treatment-related severe AEs. SD was observed in three patients, PR in one patient, and PD in the remaining patients. Three patients declined measurement of the tumor marker CA19.9. The trial reported interesting results about immune response after treatment. HF10 triggered the infiltration of macrophages and CD4^+^ and CD8^+^ T cells, as well as the activation of natural killer (NK) cells. The median progression free survival rate was six months [91,92]. A new HF10 phase I trial in patients with unresectable pancreatic cancer is in progress to assess the safety and efficacy of HF10 in combination with gemcitabine and nab-paclitaxel, as well as in combination with S-1 (tegafur/gimeracil/oteracil, TS-1^®^) (NCT03252808). Another phase I trial of T-VEC enrolled 17 patients with pancreatic cancer. The preliminary results showed that the initial doses of T-VEC (1 × 10^4^ to 1 × 10^6^ pfu/mL) were tolerated well, with the exception of two patients who experienced grade 5 AEs (not related to the T-VEC injection) without any objective response [93]. Another HSV-1 OV, Orien X010, is a recombinant hGM-CSF HSV-1 that is still ongoing in a phase I trial against pancreatic cancer (NCT01935453).

### 4.3. Pelareorep (Reolysin^®^) and Pancreatic Clinical Trials

Pelareorep (Reolysin^®^), was also tested in order to compare combined therapy with carboplatin and paclitaxel in a randomized phase II in naïve patients with metastatic pancreatic adenocarcinoma. In this trial, 36 patients who received triple combination therapy with paclitaxel/carboplatin and pelareorep were compared with 37 patients who received only combined chemotherapy with paclitaxel/carboplatin. There was no difference in the progression-free survival between the triple therapy (4.9 months) and chemotherapy groups (5.2 months). This trial demonstrated significant changes in the immunophenotypes of patients, specifically higher levels of circulating T-cells, NK cells, and proinflammatory plasma chemokines and cytokines, including IL-1β, IL-2, IL-6, IL-12p70, IL-13, IL-17A, IL-17F, IP-10, regulated on activation, normal T cell expressed and secreted (RANTES), and vascular endothelial growth factor-A (VEGFA) [94]. A separate a case report showed the first evidence of reovirus protein in a primary tumor biopsy from a pancreatic cancer patient after treatment with pelareorep and gemcitabine combination therapy [95]. In conclusion, pelareorep as a monotherapy failed to improve the progression-free survival. As a consequence, in phase II, pelareorep was combined with gemcitabine in pancreatic cancer patients. Thirty-four patients with pancreatic cancer (chemotherapy naïve patients) were enrolled in this study. On days one and eight, patients received 800 mg/m^2^ gemcitabine IV over 30 min. Moreover, on days one, two, eight, and nine, pelareorep (1 × 10^10^TCID_50_) was intravenously injected over 60 min in a three-week cycle. This combination was well tolerated, with manageable nonhematological toxicities. The study showed that one patient had partial response, 23 patients had stable disease, and five patients had a progressive disease. CA19.9 was decreased (20% from baseline) in 70% of the patients, with 10.2 months as the median overall survival. Pelareorep was replicated well in pancreatic tumors [96]. The single post-treatment biopsy using immunohistochemistry (IHC) revealed a high PD-L1 expression, which suggests that the combination of Reolysin^®^ and anti-PDL-1 may be more effective in further clinical trials. 

Currently, a phase I clinical trial of pelareorep in combination with pembrolizumab (PD-1 blockade) is ongoing in pancreatic cancer patients (NCT02620423). In addition, a phase I trial of ParvOryx is currently recruiting patients with pancreatic cancer (NCT02653313) (Table 4). In conclusion, phase I trials have been completed for only four OVs (HF10, T-VEC, ONYX-015, and pelareorep) as a monotherapy. However, the results indicate that these OVs are not yet adequate as future monotherapies. As a consequence, further clinical trials of OVs against PDA must be conducted to prove their efficacy as either a monotherapy or as part of combination therapy.

## 5. Oncolytic Viruses and Breast Cancer

Breast cancer is the most commonly diagnosed cancer among women, and the second most common cause of cancer-related deaths among women [97]. The treatment of breast cancer has advanced over the past decade, and targeted therapies have been developed, improving survival rates. However, there are still treatment-resistant cases and thus further treatment options, such as OVs, are necessary. OVs have shown promising therapeutic efficacy when used as a monotherapy in preclinical breast cancer models. Currently, different types of OVs are being investigated in clinical trials in breast cancer patients (Table 5).

### 5.1. Oncolytic Herpes Simplex Virus and Breast Cancer Clinical Trials

An investigator-initiated phase I trial of HF10 enrolled six patients with cutaneous and subcutaneous metastatic breast cancer. HF10 (1 × 10^4^:5 × 10^5^ pfu/0.5 mL/nodule) was intratumorally injected for three consecutive days. All of the patients tolerated the treatment well, and there were no reported AEs. HF10 viral inclusion bodies demonstrated a high replication selectivity in the tumor cells only. A histological examination revealed fibrosis and tumor cell death with an infiltration of CD8^+^ and CD4^+^ T-cells around tumor islets, findings that support the induction of an immune response by HF10 [98,99]. This phase I trial was concluded in 2006, and there have yet to be any phase II trials of HF10. A phase I trial of HSV-1 T-VEC enrolled 14 patients with metastatic breast cancer. The patients received an intratumoral injection of T-VEC (1 × 10^6^:1 × 10^8^ pfu/mL). All of the patients tolerated the treatment well and exhibited only grade I AEs. This therapy achieved SD in some patients, but no CR or PR. Of note, T-VEC induced necrosis of tumor cells [29].

### 5.2. Oncolytic Adenovirus and Breast Cancer Clinical Trials

A phase I trial investigated the OV adenovirus ONYX-015 in combination with etanercept (Enbrel^®^, a recombinant dimer of the human tumor necrosis factor α receptor). This study enrolled two patients with metastatic breast cancer, as well as other patients with different types of cancer. Each patient received a total of 79 injections of ONYX-015 (1 × 10^12^ vp for 4 weeks/cycle) and a subcutaneous injection of etanercept (only in cycle 1). The patients showed a transient grade I and II fever within 24 h of the ONYX-015 infusion. The patients with breast cancer showed PD with a mean survival of only 125 days. However, ONYX-015 also resulted in SD in the patients with colon cancer, demonstrating its effectiveness in colon cancer, but not in breast cancer [100].

Another clinical phase I trial using an oncolytic adenovirus, called ICOVIR-7, enrolled three patients with breast cancer. The patients received three intratumoral injections of ICOVIR-7 (1 × 10^11^ vp). One patient showed a stabilization of tumor markers, while the other patients demonstrated a negative viral genome at days 0, 2, and 7. However, at the endpoint, none of the patients showed an effective response (mild, partial, or complete response) [101].

### 5.3. Pelareorep (Reolysin^®^) and Breast Cancer Clinical Trials

A single-center, dose-escalation trial of pelareorep included two patients with breast cancer. The patients received intravenous doses ranging from 1 × 10^8^ to 3 × 10^10^ TCID_50_ every four weeks. One patient showed a PR with tumor shrinkage (34%) by RECIST criteria after five treatment cycles. Specifically, this patient showed high level of the RAS mutation, which led to the efficient viral replication and lysis of the tumor tissue. This response lasted for nine weeks [104]. A phase II clinical trial of pelareorep in combination with paclitaxel has been completed in patients with metastatic breast cancer. In this trial, seventy-four women were randomized into two arms. In arm A, 36 patients received paxlitaxel in combination with pelareorep, while 38 arm B patients received paclitaxel alone. The patients in both arms received 80 mg/m^2^ paclitaxel on days 1, 8, and 1, every 28 days, while in arm A, the patients received intravenously pelareorep 3 × 10^10^ TCID_50_ for 1 h on days 1, 2, 8, 9, 15, and 16. This trial showed that pelareorep was well tolerated. However, there was no significant difference in the progression-free survival (PFS) as a primary endpoint, and the median overall survival in arm A was significantly longer than arm B (17.4 and 10.4 months, respectively). This trial suggests that the combination of paclitaxel and pelareorep is more effective than paclitaxel alone [105].

### 5.4. Vaccinia Virus (VV_DD_) and Breast Cancer Clinical Trials

Vaccinia virus (VV_DD_) is genetically modified by the deletion of two essential genes, vaccinia growth factor and thymidine kinase, in order to acquire a tumor-only replication capacity. A trial of VV_DD_ enrolled four patients with breast cancer who were intratumorally injected with 3 × 10^7^ pfu. VV_DD_ was well tolerated and demonstrated a high selective replication in tumor cells. One patient showed grade 3 side effects, such as hemorrhage, while two patients showed an antitumor response, namely the presence of ulcerated and erythematous lesions due to viral replication [102].

### 5.5. Newcastle Disease Virus and Breast Cancer Clinical Trials

PV701, a Newcastle disease virus, was investigated in a phase I trial that enrolled two patients with breast cancer. The primary endpoint was to the maximal dosage, which could be used without toxicity. The first and second intravenously injected PV701 doses were 1 × 10^9^ pfu/m2 and 12 × 10^9^ pfu/m^2^, respectively, followed by dose increases from 24 × 10^9^ pfu/m^2^ to 120 × 10^9^ pfu/m^2^ for the third to sixth doses. PV701 was administered for an average of three completed treatment cycles. The treatment regimen was well tolerated. Mild flu-like symptoms were commonly observed after the first dose, but disappeared with repeated dosing. Moreover, dose-dependent toxicity was not observed. One of the patients showed prolonged SD for about six months [103]. Overall, several OVs were investigated in the phase I clinical trials in the patients with advanced breast cancer. The safety of these OVs was proven in these trials, but the outcomes are insufficient to warrant the use of OVs as a monotherapy against breast cancer at this time.

## 6. Future Prospective OV Clinical Trials

Although interest in using OVs in cancer therapy increased after the FDA approval of T-VEC in 2015, there are several obstacles in the development of additional oncolytic virotherapies against different cancer types. The main difficulty involves optimizing the delivery system, as the majority of OVs, except for pelareorep (Reolysin^®^), were delivered locally via direct intratumoral injection. As noted in this review, some trials attempted to optimize the OV administration using EUS guidance, a system that requires improvement in order to increase the efficacy of OV delivery against different cancer types. A second obstacle is that while the effects of OVs depend on enhancing either the innate or adaptive immune response, the immune response can also suppress viral replication, which might decrease viral oncolysis. As a consequence, the immune response needs to be balanced in favor of viral and tumor antigens. Third, pre-existing antibodies can also suppress OV efficacy when administered intravenously. Hence, coating OVs may allow for other administration options. Fourth, the strategy of combining OVs with chemotherapeutic or immunotherapeutic drugs requires further optimization, because the optimal order in which to administer these therapies is not yet clear. Finally, prognostic biomarkers for oncolytic virotherapy are needed in the future.

## Figures and Tables

**Table 1:** Genetic modification of oncolytic viruses.

Virus Classification	Oncolytic Virus	Genetic Modification	Manufacturer
**Herpes Simplex Virus-1** **(DNA Virus)**	T-VEC (talimogene laherparepvec, Imlygic^®^)	ICP34.5 deletion, ICP47 deletion, GMCSF insertion	Amgen Inc., Thousand Oaks, USA
	HF10 (canerpaturev—C-REV)	Natural deletion and insertion led to loss of expression of UL43, Ul49.5, UL55, UL56, and LAT	Takara Bio Inc., Kusatsu, Shiga, Japan
	HSV1716 (Seprehvir^®^)	ICP34.5 deletion	Virttu Biologics, Glasgow, U.K.
	G207	ICP34.5 deletion, ICP6 deletion, and LacZ insertion	Medigene, Planegg, Germany
	G47∆	ICP34.5 deletion, ICP6 deletion, ICP47 deletion, and LacZ insertion	Daiichi Sankyo Company, Tokyo, Japan
	M032	ICP34.5 deletion and IL12 insertion	Aettis, Inc., Pennsylvania, USA
	OrienX010	ICP34.5 and ICP47 deletion, and GM-CSF insertion	Orien Gene Biotechnology, Zhejiang, China
**Adenoviruses** **(DNA Virus)**	H101 (Oncorine)	E1B deletion and E3 partial deletion	Shanghai Sunway Biotech, Shanghai, China
	ONYX-015	E1B-55 KDa gene deletion	Onyx pharmaceutical, South San Francisco, USA
	ONCOS-102 (formerly named CGTG-102)	adeno ∆24-RGD-GM-CSF insertion	Targovax (Merged with ONCOS therapeutic), Oslo, Norway
	VCN-01	pRb-dependent; loaded with genes encoding PH20 hyaluronidase	VCN Biosciences SL, Barcelona, Spain
	LOAd-703	pRb-dependent; loaded with genes encoding CD40L and 4-1BBL	Lokon Pharma, Uppsala, Sweden
	ICOVIR-7	pRb-dependent adenovirus with 24-bp deletion in E1A. The fiber of the capsid has been modified with an RGD-4C motif in the HI-loop with a E2F promoter, and E1A deletion, the replication is optimized with E2F binding hairpins and contains Kozak sequence	Targovax (Merged with Oncos therapeutics), Oslo, Norway
	ICOVIR-5	Adeno ∆24-RGD, pRb-dependent adenovirus, with a deletion in 24bp in EIA with E2F promoter and contains the Kozak sequence at the E1a start codon	Institut Català d’Oncologia, Barcelona, Spain
	DNX-2401	Deletion in 24bp in EIA and RGD-motif was engineered into the fiber H-loop, enabling the virus to use α_v_β3 or α_v_β5 an integrins to enter cells	DNAtrix, Houston, USA
**Vaccinia Viruses (DNA Virus)**	Pexastimogene devacirepvec (PexaVec) (formerly named JX-594)	Thymidine kinase deletion and GM-CSF insertion	SillaJen, Busan, Korea
**Reovirus** **(RNA Virus)**	Pelareorep (Reolysin^®^)	Natural virus	Oncolytics Biotech^®^ Inc., Calgary, Canada
**Paramyxoviridae Family**	Measles virus	hNIS insertion for MV-NIS and Carcinoembryonic antigen (CEA) insertion for MV-CEA	Vyriad, Rochester, USA
	Newcastle disease virus (NDV)	Natural virus	Hadassah medical organization,
**Parvovirus** **(RNA Virus)**	Parvovirus H-1 (ParvOryx)	Natural virus	ORYX Medicine, Vaterstetten, Germany
**Picornaviruses** **(RNA Virus)**	CVA21 (Cavatak)	Natural virus	Viralytics, Sydney, Australia
	PVSRIPO	CD155/Necl5 dependent poliovirus. The internal ribosome entry site (IRES) of the poliovirus replaced with the IRES from human rhinovirus type 2 (HRV2)	Duke University, Durham, USA
**Retroviral Replicating Vectors**	TOCA51	This vector is based on murine leukemia virus (MLV) and carrying the yeast cytosine deaminase (CD) that convert 5-fluorocytosine (5-FC) into in the presence of CD, to 5-fluorouracil (5-FU).	Tocagen, San Diego, USA

The table includes the oncolytic viruses that were mentioned in this review. GM-CSF—granulocyte-macrophage colony-stimulating factor.

**Table 2:** The clinical trials of oncolytic viruses in patients with melanoma.

Oncolytic Virus	Clinical Trial Phase	Administration Route	Combination	Status	Trial No.	References
**T-VEC (Talimogene Laherparepvec)**	I	IT	----	Completed	PMID17121894	[29]
	II	IT	----	Completed	NCT00289016	[30]
	Launched	IT	----	Completed	NCT01368276, NCT00769704	[27,31,32,33,34]
	II	IT	Ipilimumab	Completed	NCT01740297	[35]
	Ib		Pembrolizumab	Completed	NCT02263508	[36]
	II	IT	Pembrolizumab	Recruiting	NCT02965716	
	I	IT	BRAF and MEK inhibitors	Recruiting	NCT03088176	
	III	IT	Dacarbazine, temozolomide	Recruiting	NCT02288897	
	II		Radiation	Recruiting	NCT02819843	
**HF10** **(Canerpaturev—C-REV)**	I	IT	----	Completed	NCT01017185	[39]
	II	IT	----	Ongoing	NCT03153085	
	II	IT	Ipilimumab	Completed	NCT02272855	[40,41]
	II	IT	Nivolumab	Recruiting	NCT03259425	
**OrienX010**	I	IT	----	Recruiting	NCT03048253	
**Oncos-102**	I	IT	Cyclophosphamide pembrolizumab	Recruiting	NCT03003676	
**ICOVIR-5**	I	IT	----	Completed Not reported	NCT01864759	
**Pexa Vec/JX-594**	I	IV	----	Completed	PMID10505851	[54]
	II	IT	----	Completed	NCT00429312	[55]
**Coxsackievirus A21 (CVA21)** **Cavatak**	I	IT	----	Completed	NCT00438009	[44,45]
	II	IT	----	Completed	NCT01227551	[46]
	I	IT	Ipilimumab	Recruiting	NCT02307149	[47,48]
	I	IT	Pembrolizumab	Recruiting	NCT02565992	
**Pelareorep** **(Reolysin^®^)**	I	IV	----	Completed	PMID: 18981012	[51]
	II	IV	----	Completed	NCT00651157	[52]
	II	IV	Carboplatin/paclitaxel	Completed	NCT00984464	[53]

Abbreviation: IT—intratumoral route; IV—intravenous.

**Table 3:** The clinical trials of oncolytic viruses in patients with glioma.

Oncolytic Virus	Phase	Administration Route	Combination	Status	Trial No.	Reference
**HSV1716** **Seprehvir^®^**	I	IT	----	Completed	PMID10845724	[60]
	Ib	IT	----	Completed	PMID11960316, PMID15334111	[61,62]
	II	IT	----	Completed Not reported	NCT02031965	
**G207**	I	IT	----	Completed	NCT00028158	[64]
	Ib	IT	----	Completed	NCT00157703	[65,66]
	I	IT	5Gy radiation dose	Completed	NCT00157703.	[67]
**G47∆**	I	IT	----	Completed Not reported		[69]
	II	IT	----	Ongoing	UMIN000011636	[69]
**MO32**	I	IT		Ongoing	NCT02062827	[58]
**rQNestin**	I	IT		Recruiting	NCT03152318	
**ONYX-015**	I	IT	----	Completed	PMID15509513	[70]
**Adeno-Delta-24-RGD** **(DNX-2401)**	I	IT	----	Completed		[71,72]
	I		temozolomide	Ongoing	NCT01956734	
	I		interferon-γ	Ongoing	NCT02197169	
**Pelareorep** **(Reolysin^®^**)	I	IV	----	Completed	NCT00528684	[73]
	I	IV	----	Completed	PMID: 24553100	[74]
**Newcastle Disease Virus (NDV)**	I/II	IT	----	Completed	NCT01174537	[75]
**Parvovirus H-1 (ParvOryx)**	I	IT and IV	----	Completed	NCT01301430	[76]
**Measles virus CEA**	I	IT	----	Ongoing	(NCT00390299)	
**Poliovirus PVSRIPO**	I	IT	----	Completed	(NCT01491893)	[77]
**Toca 511**	I	IT	----	Ongoing	(NCT02576665)	
**Vaccinia virus TG6002**	I	IT	----	Ongoing	(NCT03294486)	

**Table 4:** The clinical trials of oncolytic viruses in patients with pancreatic cancer.

Oncolytic Virus	Clinical Trial Phase	Administration Route	Combination	Status	Trial No	Reference
**T-VEC (Talimogene laherparepvec)**	I	IT	----	Completed	NCT00402025	[93]
	I	IT	--	Recruiting	NCT03086642	
**HF10** **(canerpaturev, C-REV)**	IIT	IT	----	Completed	PMID29801474	[91,92]
	I	IT	Nab-Paclitaxel + Gemcitabine/S-1	Ongoing	NCT03252808	
**OrienX010**	I	IT	----	Ongoing	NCT01935453	
**ONYX-015**	I	IT	----	Completed	PMID11313805	[89]
	II	IT	Gemcitabine	Completed	PMID12576418	[90]
**VCN-01**	I	IT &IV	Nab-Paclitaxel/Gemcitabine	Recruiting	NCT02045589	
**LOAd703**	I	IT	Gemcitabine	Recruiting	NCT02705196	
**Parvovirus H-1 (ParvOryx)**	I	IT & IV	----	Recruiting	NCT02653313	
**Pelareorep** **(Reolysin^®^**)	II	IV	Carboplatin/paclitaxel	Completed	NCT01280058	[94]
	II	IV	Gemcitabine	Completed	NCT00998322	[95]
	I	IV	Pembrolizumab	Ongoing	NCT02620423	

**Table 5:** The clinical trials of oncolytic viruses in patients with breast cancer.

Oncolytic Virus	Clinical Trial Phase	Administration Route	Combination	Status	Trial No.	Reference
**T-VEC (Talimogene laherparepvec)**	I	IT	----	Completed	PMID17121894	[29]
**HF10 (canerpaturev—C-REV)**	IIT	IT	----	Completed	PMID16865590	[98,99]
**ONYX-015**	I	IT	Enbrel	Completed	PMID17704755	[100]
**ICOVIR-7**	I	IT	----	Completed	PMID20501623	[101]
**Vaccinia Virus (VV_DD_)**	I	IT	----	Completed	PMID25292189	[102]
**PV701**		IV	----	Completed	PMID16638865	[103]
**CVA21**	I	IT	----	Completed Not reported	NCT00636558	
**Measles Virus (MV)-NIS**	I	IT	----	Ongoing	NCT01846091	
	I	IT	----	Recruiting	NCT01376505	
**Pelareorep** **(Reolysin^®^)**	I	IV	----	Completed	PMID19572105	[104]
	II	IV	Paclitaxel	Completed	NCT01656538	[105]
